# IFITM3-expressing *Lactobacillus plantarum* (HA-r-LAB) as a probiotic vector for targeted immunotherapy against TGEV

**DOI:** 10.1186/s13567-025-01669-8

**Published:** 2025-12-29

**Authors:** Binghan Bai, Maokun Zhu, Ling Zhang, Lixin Wen, Xiaomin Yuan

**Affiliations:** 1 College of Veterinary Medicine, Hunan Agricultural University (HUNAU), Changsha, 410128 Hunan China; 2 Changsha Green Leaf Bio Technology Co., Ltd., Changsha, 410119 Hunan China; 3 Institute of Yunnan Circular Agricultural Industry, Puer, 665000 Yunnan China

**Keywords:** Lactic acid bacteria, hyaluronic acid, IFITM3 immunotherapy, TGEV, probiotic

## Abstract

*Lactiplantibacillus plantarum*, recognized for its potent probiotic functionality and advanced drug delivery capability, has emerged as a versatile platform for the heterologous expression of bioactive therapeutic proteins. In this study, we genetically engineered *L. plantarum* to express interferon-induced transmembrane protein 3 (IFITM3), an immunomodulatory protein that exhibits extensive antiviral efficacy by predominantly obstructing viral entry. To enhance gastrointestinal resilience and systemic bioavailability, the recombinant strain was encapsulated with hyaluronic acid (HA-r-LAB) through a precise nanoencapsulation methodology, generating stable nanoparticle-like constructs. In a piglet model challenged with transmissible gastroenteritis virus (TGEV), a prototypical coronavirus, oral administration of HA-r-LAB expressing IFITM3 substantially decreased viral titres and alleviated clinical manifestations. Furthermore, biochemical analyses confirmed a direct molecular interaction between IFITM3 and the TGEV nucleocapsid (N) protein, suggesting an unprecedented antiviral mechanism. These findings underscore the potential of HA-r-LAB as a probiotic-based nanodelivery vector for antiviral proteins and lay the groundwork for the development of combinatorial therapeutic strategies targeting coronaviruses.

## Introduction

Lactic acid bacteria (LAB), characterized by their Gram-positive nature and low GC content, are well known for fermenting carbohydrates into lactic acid [1, 2]. LAB have traditionally been employed in the fermentation of milk, vegetables, and meat products [3], and their therapeutic potential in managing gastrointestinal and systemic disorders has garnered significant attention [4, 5]. Recent advancements have led to the development of recombinant LAB (r-LAB), which are engineered to deliver therapeutic proteins directly to mucosal surfaces [6]. These characteristics have paved the way for applications in food science, healthcare, and agriculture.

Despite these promising features, the clinical application of oral probiotic therapies is hampered by the limited persistence of probiotics in the gastrointestinal tract (GIT) due to harsh conditions such as gastric acidity, bile salts, and digestive enzymes [7–9]. Hyaluronic acid (HA), a naturally occurring anionic polysaccharide known for its biocompatibility and modifiability [10, 11], possesses molecular weight–dependent anti-inflammatory and immunomodulatory properties [12]. These features have prompted the exploration of HA-based materials for encapsulating and delivering probiotics [13]. In this study, we developed an HA hydrogel to encapsulate LAB, thereby increasing their survival and therapeutic potential in the GIT [14].

Coronaviruses are notorious for their genetic variability and cross-species transmission, posing major challenges for infectious disease management [15]. Among them, transmissible gastroenteritis virus (TGEV)—a coronavirus with a genome of approximately 28.5 kilobases—poses a significant threat to the global swine industry by causing severe gastroenteritis in pigs [16]. The clinical manifestations of TGEV infection include vomiting, severe diarrhea, and dehydration [17], with mortality rates reaching 100% in piglets under two weeks of age [18]. Despite widespread vaccination and conventional preventive measures, TGEV continues to inflict substantial economic losses on swine production worldwide [19]. Therefore, novel therapeutic strategies against TGEV and its complications are urgently needed [20].

Interferon-induced transmembrane protein 3 (IFITM3) is a key antiviral protein that prevents the fusion of enveloped viruses with host cell membranes, thereby inhibiting infections such as influenza, dengue, and Zika [21]. Its abundant localization in endosomal membranes renders IFITM3 particularly effective against viruses that enter cells via endocytosis [22]. However, its efficacy is reduced against viruses that utilize plasma membrane fusion (e.g., Sendai virus) [23, 24] and is partially effective against viruses capable of dual entry mechanisms (e.g., human metapneumovirus) [25]. These observations suggest that the antiviral role of IFITM3 may vary among different viruses, necessitating a detailed investigation of its interaction with TGEV, which appears to employ dual entry pathways [26, 27].

In this study, we employed LAB as a delivery vehicle for IFITM3 and enhanced its gastrointestinal survival through an HA protective layer. We aimed to evaluate whether HA-encapsulated recombinant Lactobacillus (HA-r-LAB) could serve as an effective oral delivery vehicle for IFITM3 in a porcine model [28]. We further sought to investigate the antiviral potential of IFITM3 at both the clinical and molecular levels, including its interaction with the TGEV nucleocapsid protein and its impact on intestinal inflammation and tissue integrity.

## Materials and methods

### Construction of r-LAB

**Figure 1:**
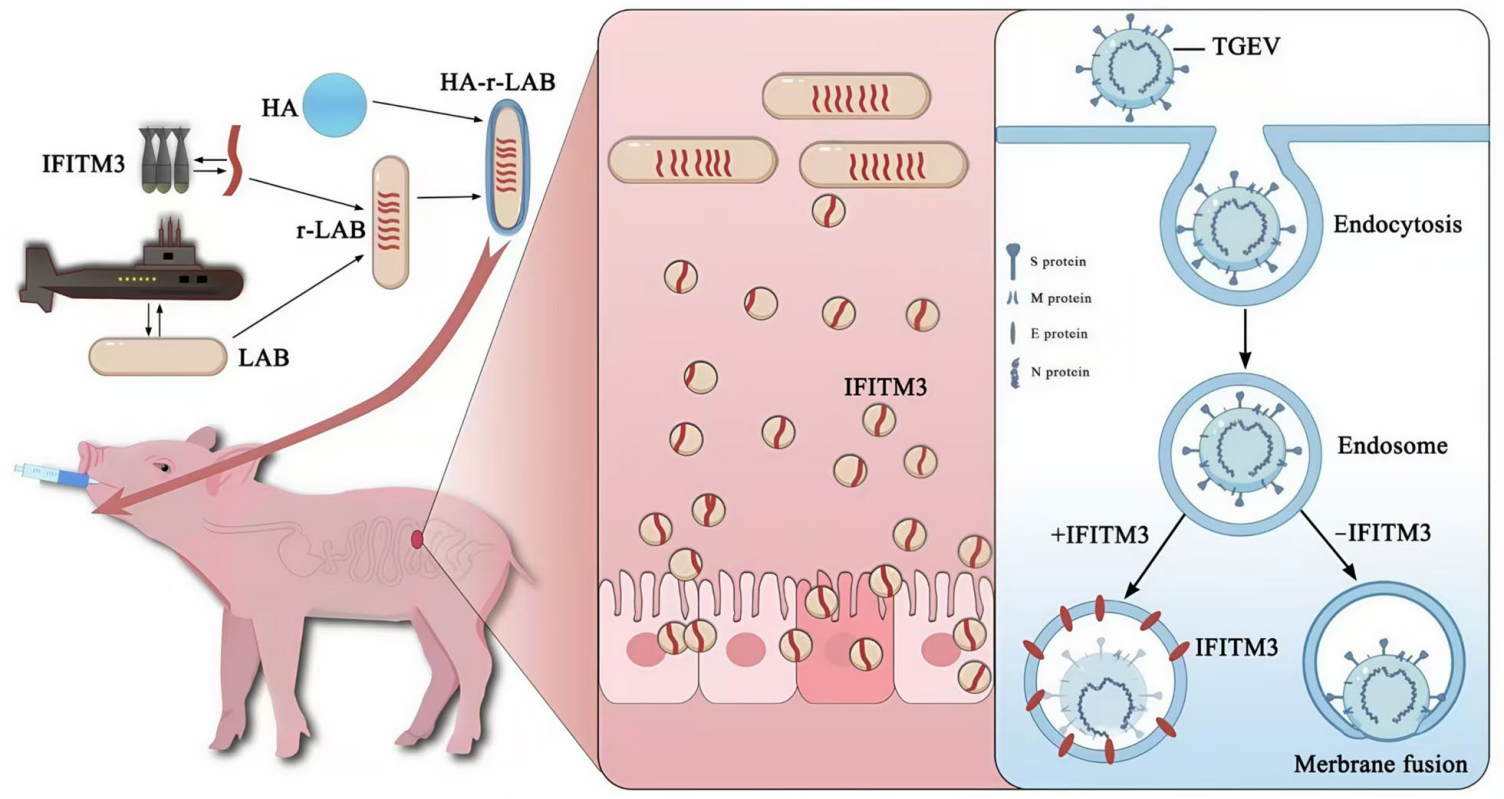
**Mode of action diagram.** Schematic representation of the experimental strategy used to study the antiviral mechanism of HA-encapsulated recombinant *L. plantarum* (HA-r-LAB) expressing porcine IFITM3 protein against TGEV infection. HA-r-LAB are orally administered to pigs, allowing the recombinant bacteria to reach the intestinal tract. Upon colonization, r-LAB release the IFITM3 protein, which is taken up by intestinal epithelial cells. IFITM3 localizes to endosomal membranes and disrupts the membrane fusion process required for TGEV entry, thereby inhibiting viral infection. The right panel illustrates the endocytic entry of TGEV and how IFITM3 blocks membrane fusion in endosomes.

*Lactobacillus plantarum* CICC6240 and the expression plasmid pMG36e were kindly provided by Changsha Green Leaf Bio Technology Co., Ltd. Total RNA was extracted from porcine alveolar macrophages (3D4/21 cells) via the TRIzol method (TransGen, China) and reverse transcribed into cDNA. A 498 bp fragment of the porcine IFITM3 gene—flanked by a FLAG tag at the 5′ end and a 6 × His tag at the 3′ end—was amplified by PCR. This fragment was inserted into the pMG36e vector via double digestion to generate the pMG36e IFITM3 recombinant plasmid. Competent CICC6240 cells were prepared using the calcium chloride method, and electroporation was used to introduce the plasmid, yielding the r-LAB strain (see Figure 1).

### Construction of HA-r-LAB

According to the experimental requirements, hyaluronic acid sodium (HA) with a molecular weight of 5 kDa was selected. To 20 mL of deionized water, 0.1 g of accurately weighed hyaluronic acid sodium was added, and the mixture was stirred for 30 min via a magnetic stirrer to ensure complete dissolution, resulting in a 0.5% HA solution. The cultured recombinant lactic acid bacteria (r-LAB) were suspended in the aforementioned HA solution, gently stirred or shaken to allow HA to evenly coat the surface of the r-LAB. The mixture was then incubated at room temperature for 30 min to facilitate the coating process. After coating, the excess HA solution was removed by centrifugation (4000 × *g*, 10 min). The r-LAB were washed with deionized water, and the centrifugation and washing steps were repeated three times to remove unbound HA. Finally, the HA-r-LAB complex was resuspended in 10 mL of deionized water for further use.

### Observation by scanning electron microscopy (SEM)

The HA-r-LAB complex was placed on a conductive sample holder (e.g., copper plate) and dried in a desiccator for 24 h to remove moisture. To enhance contrast, the sample was coated with gold using sputtering, and the parameters were confirmed with laboratory staff.

The SEM sample chamber was opened, the holder was installed, and the chamber was evacuated to 10⁻⁶ Torr. The SEM was powered on and self-checked, and the software was launched. The settings were as follows: 15 kV acceleration voltage, 100 Hz scanning speed, and 100 pA probe current. Coarse and fine focusing were performed manually or via software, and astigmatism was corrected. The sample position was adjusted using a mouse, and the image contrast and gain were fine-tuned. The final image was saved in TIFF format.

### Growth characteristics of rLAB

CICC6240 and HA r LAB were inoculated into MRS media at a 1:100 dilution and incubated at 30 °C for 24 h. The optical density (OD_600_) was measured every 2 h during the first 12 h and every 6 h thereafter to generate growth curves. Additional incubations at 23 °C, 30 °C, and 37 °C were performed to assess temperature-dependent growth. Plasmid stability was evaluated by culturing CICC6240/pMG36e IFITM3 for 30 generations and assessing colony formation on chloramphenicol-containing plates.

### Simulated gastrointestinal fluid tolerance assay

Simulated gastric fluid (SGF) was prepared by dissolving pepsin (0.32 mg/mL) and NaCl (0.9%) in distilled water and adjusting the pH to 2.0 with HCl. The mixture was incubated at 37 °C. Simulated intestinal fluid (SIF) was prepared by adding pancreatin (1 mg/mL) and bile salts (3 mM) to distilled water and adjusting the pH to 7.4 with NaHCO₃. The mixture was also incubated at 37 °C. The bacterial suspensions were divided into four groups: the mock group (untreated control, cultured directly in MRS medium); the SGF group (treated with SGF for 1 h at 37 °C); the SIF group (treated with SIF for 2 h at 37 °C); and the SGF + SIF group (treated sequentially with SGF for 1 h, followed by SIF for 2 h at 37 °C to simulate gastrointestinal transit conditions). Equal volumes of bacterial cultures were added to each treatment group. After incubation, the samples were serially diluted tenfold (from 10⁻^1^ to 10⁻⁶), plated on MRS agar, and incubated anaerobically at 37 °C for 48 h. The number of colony-forming units (CFU/mL) was then counted to evaluate bacterial viability.

### Intestinal colonization assay

Kunming mice (SPF grade, purchased from SJA Laboratory Animal Co., Ltd., China) were used to establish the colonization model. The mice were orally administered bacterial strains (CICC 6240 or HA-r-LAB) to assess colonization in the gastrointestinal tract. At 24 to 48 h post-gavage, the mice were euthanized. The duodenum, jejunum, and ileum were carefully excised under sterile conditions. Each intestinal segment was cut into small pieces, homogenized in sterile PBS, and subjected to tenfold serial dilutions. The diluted samples were plated on MRS agar and incubated anaerobically at 37 °C for 48 h. Colonization levels are expressed as colony-forming units (CFUs) per gram of tissue.

### Piglet challenge experiment

One-week-old Large White piglets (conventional grade, obtained from SJA Laboratory Animal Co., Ltd., China) were prefeed with sterile formula milk for 7 days under controlled conditions. The piglets were subsequently orally administered 200 µL of PBS (control), 200 µL of CICC 6240 suspension, or 200 µL of HA-r-LAB suspension once daily for 3 consecutive days. On day 4, the piglets were orally challenged with 10 mL of TGEV (TCID_50_ = 10^8^/mL; provided by the Laboratory of Animal Disease Molecular and Immunology, Hunan Agricultural University). Surviving piglets were euthanized on day 12. All the piglets were confirmed to be free from common enteric pathogens, including transmissible gastroenteritis virus (TGEV), porcine epidemic diarrhea virus (PEDV), and porcine rotavirus, as verified by RT‒PCR prior to the experiment.

### Pathological tissue sections

Piglets were dissected, and the brain, lung, and liver were collected and fixed in 4% paraformaldehyde for 2 days. The tissues were then dehydrated through a graded ethanol series, cleared with xylene, and embedded in paraffin wax after soaking in molten paraffin for 2 h. The embedded tissues were cut into 5 µm thin slices, placed on slides, and dried, after which the sections were stained with haematoxylin‒eosin (H&E). Finally, the sections were sealed with neutral resin and coverslips, air-dried, and observed with an OLYMPUS CX33RTFS2 microscope at 100 × magnification.

### RNA extraction and qPCR

Tissues (brain, lung, spleen, and liver) were processed sing the Animal Tissue DNA Extraction Kit (TianGen, China), and absolute quantitative PCR was performed with a TGEV standard plasmid (R^2^ = 0.996). Total RNA was extracted with TRIzol and reverse transcribed (using 1 µg of RNA), and real-time PCR was conducted with 1 µL of cDNA in a 10 µL reaction mixture with SYBR Green master mix (Vazyme, China). Relative gene expression was calculated using the 2^−ΔΔCt^ method, with GAPDH used as the reference gene. The primers used for TGEV, IL-1β, IL-10, TNFα, IL-6, and GAPDH were synthesized by Beijing Tsingke Biotech Co., and the primer sequences are presented in Table 1.

**Table 1:** **Sequences of primers used in this study**

PCR	
IFITM3-F	GGGGTACCCCATGGATTACAAGGATGACGACGATAAGAATTGCGCTTCCCAG
IFITM3-R	GCTCTAGATTACATCATCACCATCACCACGTAGCCTCTGTAATC
qPCR	
TGEV-F	GACCCCGAGGACGAGTTCA
TGEV-R	ACGCCATAGTTGGGTCCATT
IL-1β-F	GAAATGCCACCTTTTGACAGTG
IL-1β-R	TGGATGCTCTCATCAGGACAG
IL-10-F	GCTCTTACTGACTGGCATGAC
IL-10-R	CGCAGCTCTAGGAGCATGTG
TNF-α-F	ATGAGCACTGAGAGCATGATCCG
TNF-α-R	CCTCGAAGTGCAGTAGGCAGA
IL-6-F	TCCTTCTTGGGACTGATGCT-
IL-6-R	GCCAGTCTTCTTCCAGTTC
GAPDH-F	TTCACCACCATGGAGAAGGC
GAPDH-R	GGCATGGACTGTGGTCATGA

### Comprehensive clinical and histopathological scoring criteria for TGEV-infected piglets

The detailed evaluation criteria are presented in Tables 2, 3, 4, 5.

**Table 2:** **Scoring criteria for the degree of diarrhea in piglets**

Score	Description of diarrhea
0	Normal faeces, no diarrhoea
1	Slightly soft faeces, but formed
2	Soft and partially watery faeces, not entirely watery
3	Mainly watery faeces, with a small amount of undigested feed
4	Entirely watery faeces, no solid content, may contain blood or mucus

**Table 3:** **Scoring of changes in piglet mental state**

Score	Description of mental state
0	Active and lively, responsive to stimuli, no abnormal behavior
1	Slightly less active, but responsive to external stimuli
2	Reduced activity, slow response to external stimuli
3	Lying down most of the time, occasional response to stimuli
4	Lying down for extended periods, almost no response to stimuli

**Table 4:** **Scoring of intestinal pathological changes in piglets**

Score	Description of pathological changes
0	No visible pathological changes, intact tissue structure
1–2	Mild epithelial cell damage, slight inflammatory cell infiltration
3–4	Moderate villous atrophy, obvious inflammatory cell infiltration
5–6	Significant villous atrophy, crypt hyperplasia, widespread inflammatory cell infiltration
7–8	Extensive villous loss, pronounced crypt hyperplasia, severe inflammatory response
9–10	Complete villous disappearance, severe epithelial damage, accompanied by extensive inflammatory cells and tissue necrosis

**Table 5:** **Piglet post-infection symptom scoring**

Piglet Group	Days post TGEV Infection							
	1	2	3	4	5	6	7	8
Mock	-a/-b/-c/-d	-a/-b/-c/-d	-a/-b/-c/-d	-a/-b/-c/-d	-a/-b/-c/-d	-a/-b/-c/-d	-a/-b/-c/-d	-a/-b/-c/-d
PBS	-a/-b/-c/-d	-a/-b/-c/-d	+ a/ + + b/-c + / + d	+ + a/ + + b/ + + + c/ + + + d	+ + + a/ + + b/ + + + c/ + + + + + d	+ + + a/ + + + b/ + + + c/ + + + d	+ + + a/ + + + b/ + + + c/ + + + d	ND
CICC6240	-a/-b/-c/-d	-a/-b/-c/-d	+ + a/ + + b/ + c/ + d	+ + a/ + + b/ + + c/ + + + d	+ + a/ + + b/ + + + c/ + + + d	+ + + a/ + + + b/ + + + c/ + + + d	+ + + a/ + + + b/ + + + c/ + + + d	+ + + a/ + + + b/ + + + c/ + + + d
HA-r-LAB	-a/-b/-c/-d	-a/-b/-c/-d	+ a/-b/ + c/ + d	+ a/ + b/ + c/ + d	-a/-b/ + c/ + d	+ a/-b/ + c/ + d	-a/-b/-c/-d	-a/-b/-c/-d

^a^Severity of diarrhea:—no diarrhea; + mild diarrhea; +  + moderate diarrhea; +  +  + severe diarrhea; ND: not determined due to the death of this piglet.

^b^Degree of appetite loss:—normal appetite; + slight loss of appetite, +  + moderate loss of appetite; +  +  + no appetite.

^c^Loss of mental stability: mental stability; + mentally unstable.

^d^TGEV detection in faeces by RT‒PCR:—negative; + positive.

### Coimmunoprecipitation (Co-IP) assays and western blotting analyses

293FT cells, the TGEV-N and IFITM3 plasmids, were prepared by our laboratory. 293FT cells were cultured in DMEM (Gibco, USA) supplemented with 1% penicillin–streptomycin solution (Gibco, USA) and 10% FBS (Gibco, USA). 293FT cells were grown to 90% confluence at 37 °C in a humidified 5% CO_2_ incubator, passaged and subcultured with 0.25% trypsin (Gibco, USA). At 48 h after transfection, the 293FT cells were lysed for 20 min at 4 °C in RIPA buffer after being rinsed with phosphate-buffered saline (PBS). The cell lysate supernatants were treated for an overnight period at 4 °C with DynabeadsTM magnetic beads (Invitrogen, USA) and either an anti-Flag (Abcam) or anti-HA antibody (Elabscience). r-LAB were induced with 50 µg/mL nisin (MCE) for 12 h, followed by treatment with 0.1 mg/mL lysozyme (Solarbio, China) at 37 °C for 1 h. Total protein was extracted from the jejunum and ileum of 26-day-old piglets assigned to four experimental groups: the mock group, the PBS-treated group, the CICC6240-treated group, and the HA-r-LAB-treated group. The latter three groups were all challenged with TGEV. The BCA method was used to determine the protein concentration for western blot analysis. After that, the samples were subjected to SDS‒PAGE and transferred to polyvinylidene difluoride (PVDF) membranes (Millipore, Billerica, MA, USA). The PVDF membranes were blocked with 5% skim milk in PBST with 0.1% Tween 20 for 3 h and then incubated with primary antibodies at 37 °C for 2 h and with horseradish peroxidase-conjugated secondary antibody at 37 °C for 45 min. Following a 30-min PBST wash, the target protein bands were identified by an imaging laboratory system (Bio-Rad ChemiDoc XRS +).

### Protein–protein interaction prediction and structural visualization

The three-dimensional structures of porcine IFITM3 and the TGEV nucleocapsid (N) protein were predicted using AlphaFold3 [29]. Protein–protein docking was performed on the basis of the structural prediction results to explore potential interaction sites. The predicted docking complex was subsequently visualized and analysed via PyMOL (version 3.1.4.1; Schrödinger, LLC).

### Data analysis of RNA-Seq

PK15 cells were cultured in 6-well plates for 24 h at 37 °C with 5% CO_2_ and then transfected with the TGEV-N plasmid. The cells were washed with 1 × PBS, fresh media was added, and the cells were maintained at 37 °C with 5% CO_2_. Total RNA was collected with TRIzol at 48 h. RNA quantity and quality were assessed via the RNA Nano 6000 Assay Kit of the Bioanalyzer 2100 system (Agilent Technologies, CA, USA). All the RNA samples were high quality, as determined via the use of poly-T oligo-attached magnetic beads. The RNA-seq libraries were generated via the AMPure XP system (Beckman Coulter, Beverly, USA). The quality and quantity of the libraries were assessed on an Agilent Technologies 2100 Bioanalyzer using a high-sensitivity DNA chip.

### Statistical analysis

Multiple t tests in GraphPad Prism 8 were used to analyse experimental data between groups. All the experiments were repeated 3 or more times. *P* < 0.05 was considered statistically significant.

## Results

### Construction and physiological characterization of HA-r-LAB

**Figure 2:**
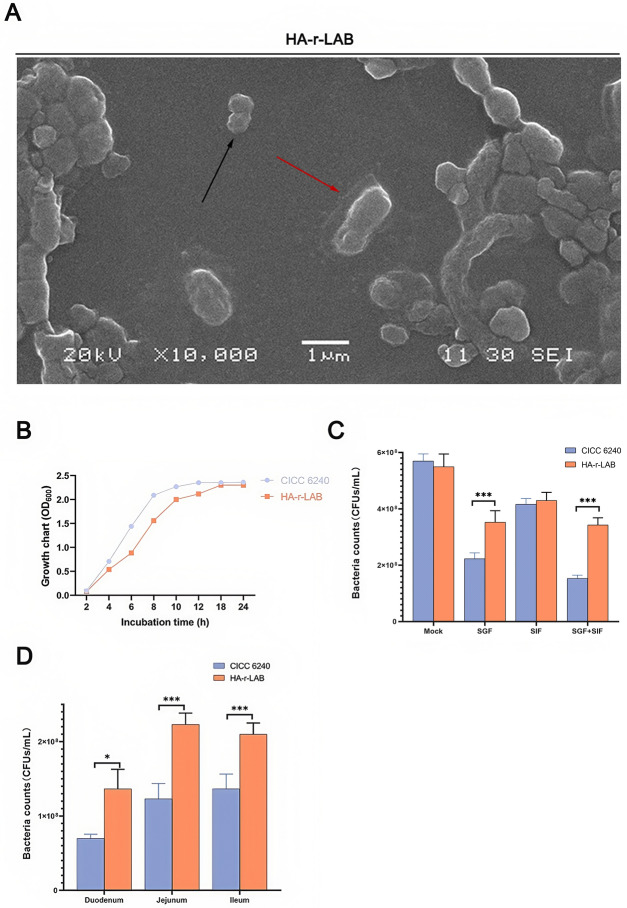
**Evaluation of HA-r-LAB bacterial survival and growth.** **A** Scanning electron microscopy (SEM) images depict the morphological features of HA-r-LAB, highlighting the differences in surface texture and structure with and without the hyaluronic acid (HA) coating across various magnifications. r-LAB are marked with black arrows, and HA-r-LAB are marked with red arrows. **B** Growth curve comparing CICC 6240 and HA-r-LAB over a 24-h period, as measured by optical density (OD_600_). These data illustrate that HA-r-LAB sustains growth kinetics comparable to those of the unmodified CICC 6240 strain. **C** This bar chart presents bacterial counts for CICC 6240 and HA-r-LAB under different simulated gastrointestinal fluid (SGF) conditions: mock (no treatment), SGF, SGF with pepsin (SIF), and SGF with SIF (SGF + SIF). The results, expressed as colony-forming units (CFUs) per milliliter, demonstrated that HA-r-LAB improved survival, particularly in the presence of bile salts. **D** Bacterial counts in the duodenum, jejunum, and ileum of piglets are shown for both CICC 6240 and HA-r-LAB. The bar chart, which represents CFUs per milliliter, indicates that HA-r-LAB has a significantly greater colonization ability in all three sections of the gastrointestinal tract than CICC 6240 does. The data were repeated three or more times and are expressed as the means ± SDs. *p < 0.05; **p < 0.01; ***p < 0.001.

Scanning electron microscopy revealed that HA-coated bacteria presented a distinct surface layer compared with the smooth surface of uncoated CICC6240 (Figure 2A). The growth curves over 36 h did not differ between HA-r-LAB and CICC6240 (Figure 2B), and plate counts confirmed the excellent biocompatibility of the HA coating (Figure 2C). Under simulated extreme gastrointestinal conditions, HA-r-LAB presented significantly higher survival rates than did uncoated bacteria (Figure 2D).

### HA-r-LAB alleviates clinical symptoms in TGEV-infected piglets

**Figure 3:**
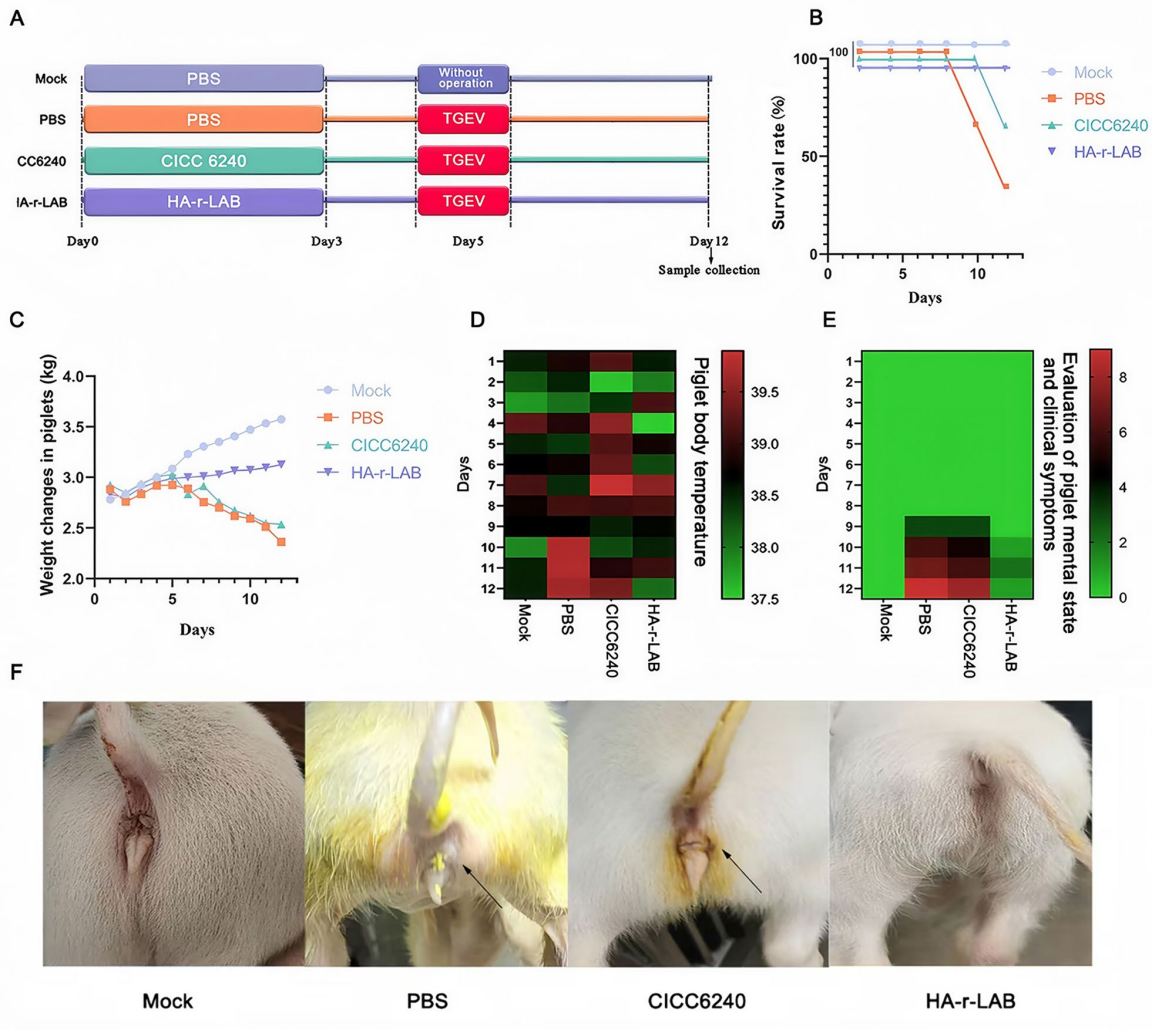
**Therapeutic efficacy of HA-r-LAB in TGEV-infected piglets.** **A** Experimental timeline showing the administration of different treatments to piglets, including mock, PBS, CICC 6240, and HA-r-LAB, with TGEV infection applied on day 3. **B** Survival rate of piglets after TGEV infection across different treatment groups, with HA-r-LAB improving the survival rate compared with the other groups. **C** Weight change percentage of piglets over time, indicating that HA-r-LAB treatment helps maintain body weight better than other treatments do. **D** Heatmap representing piglet body temperatures across different treatment groups, with HA-r-LAB maintaining temperatures closer to normal than those in the PBS group. **E** Evaluation of piglet mental state and clinical symptoms over the course of the experiment, demonstrating that HA-r-LAB treatment leads to better overall health and fewer symptoms. **F** Photographs of piglets showing clinical symptoms such as diarrhea post TGEV infection, with HA-r-LAB treatment resulting in visibly healthier piglets than those in the PBS and CICC 6240 groups. The symptoms of piglet diarrhea are marked with black arrows.

A comprehensive immunoprotection study was conducted using piglets, with the animals randomized into different treatment groups (Figure 3A). The HA-r-LAB group showed a higher survival rate compared to both the negative control and mock-treated groups (Figure 3B). In addition, piglets receiving HA-r-LAB experienced a markedly reduced decline in body weight—a critical indicator of disease progression (Figure 3C). Clinical assessments of body temperature and mental status in the HA-r-LAB group were comparable to those in the mock-treated group (Figure 3D–E), and on-site photographs revealed a reduction in diarrhea symptoms (Figure 3F). Collectively, these results underscore the robust protective effects of HA-r-LAB against TGEV infection. The specific clinical symptom scores of the piglets can be found in Table 5. This table provides a detailed breakdown of the clinical symptoms observed in the piglets, allowing for a comprehensive assessment of their health status.

### HA-r-LAB ameliorates pathological manifestations in TGEV-infected piglets

**Figure 4:**
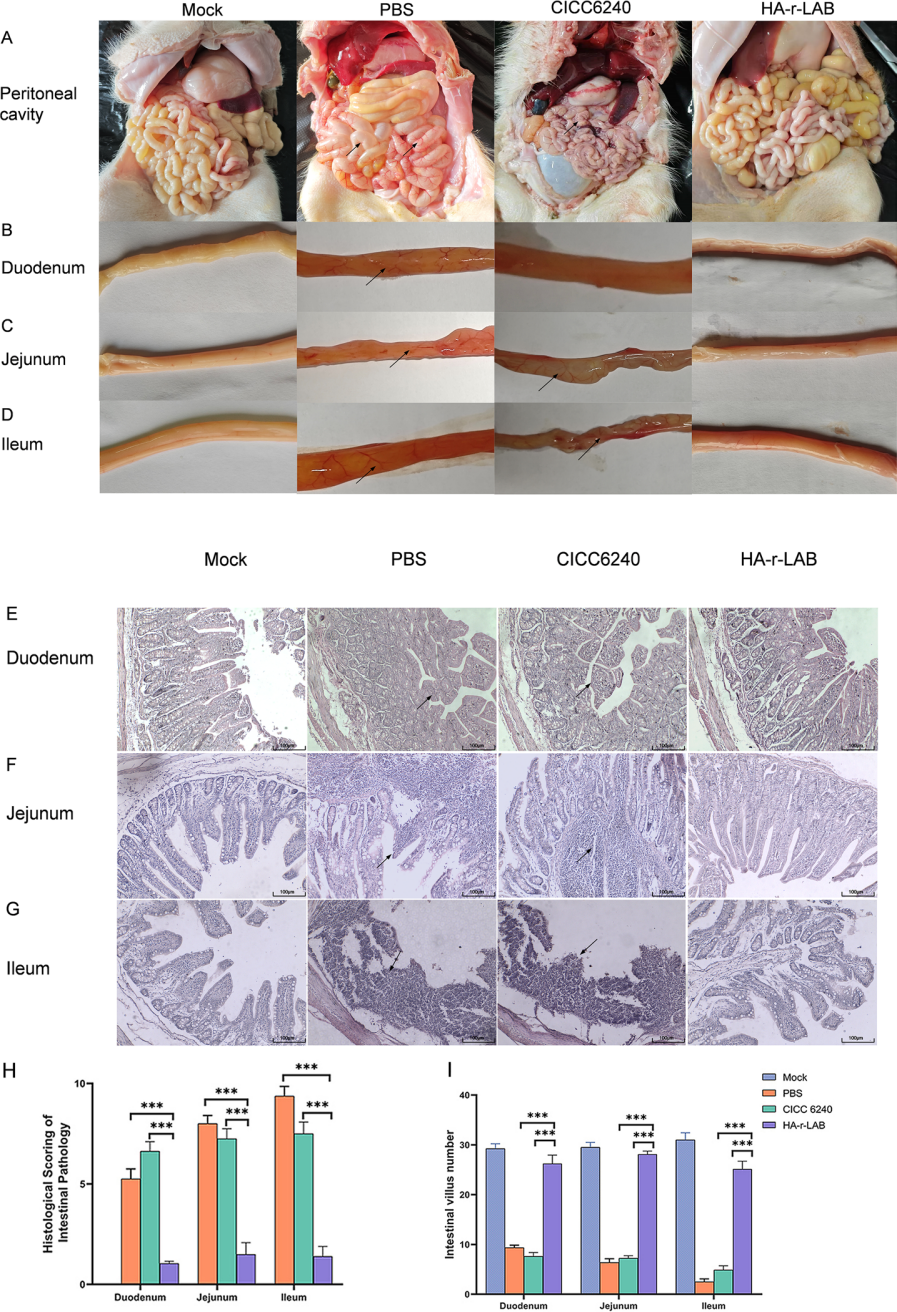
**Histopathological assessment and morphological observations of TGEV-infected piglets treated with different regimens.** **A**–**D** Gross morphology: After TGEV infection, the PBS/CICC6240 groups presented intestinal thinning, transparency, and hemorrhage (marked with black arrows); HA-r-LABs presented minimal damage. **E**–**G** Histopathology (H&E): The mock group retained normal villus/crypt architecture. The PBS/CICC6240 groups displayed villus atrophy, mucosal disruption, and shallow crypts (arrows); HA-r-LAB preserved tissue integrity. **H** Pathology scores: The PBS/CICC6240 groups had significantly higher scores than the HA-r-LAB groups did in the duodenum, jejunum, and ileum. **I** Villus count: Mock/HA-r-LAB groups maintained normal villus numbers; the PBS/CICC6240 groups presented significant reductions (arrows). The data were repeated three or more times and are expressed as the means ± SDs. *p < 0.05; **p < 0.01; ***p < 0.001.

Necropsy of the experimental piglets revealed significant differences between the treatment groups (Figure 4A). The PBS group exhibited markedly thinned, translucent intestinal walls with dilated blood vessels—especially in the jejunum and ileum—indicative of severe structural disruption. In contrast, piglets treated with HA-r-LAB displayed minimal pathological changes (Figures 4B–D), with anatomical diagrams confirming preserved intestinal integrity. Histopathological analysis revealed severe villus shedding in the PBS group (Figures 4E–G), whereas the HA-r-LAB group maintained villus counts similar to those of the mock-treated group. Statistical analysis confirmed that HA-r-LAB significantly reduced villus shedding and preserved villus architecture, which is essential for nutrient absorption and gut health (Figures 4H–I).

### HA-r-LAB modulates cytokine expression and interferes with TGEV-host interactions

**Figure 5:**
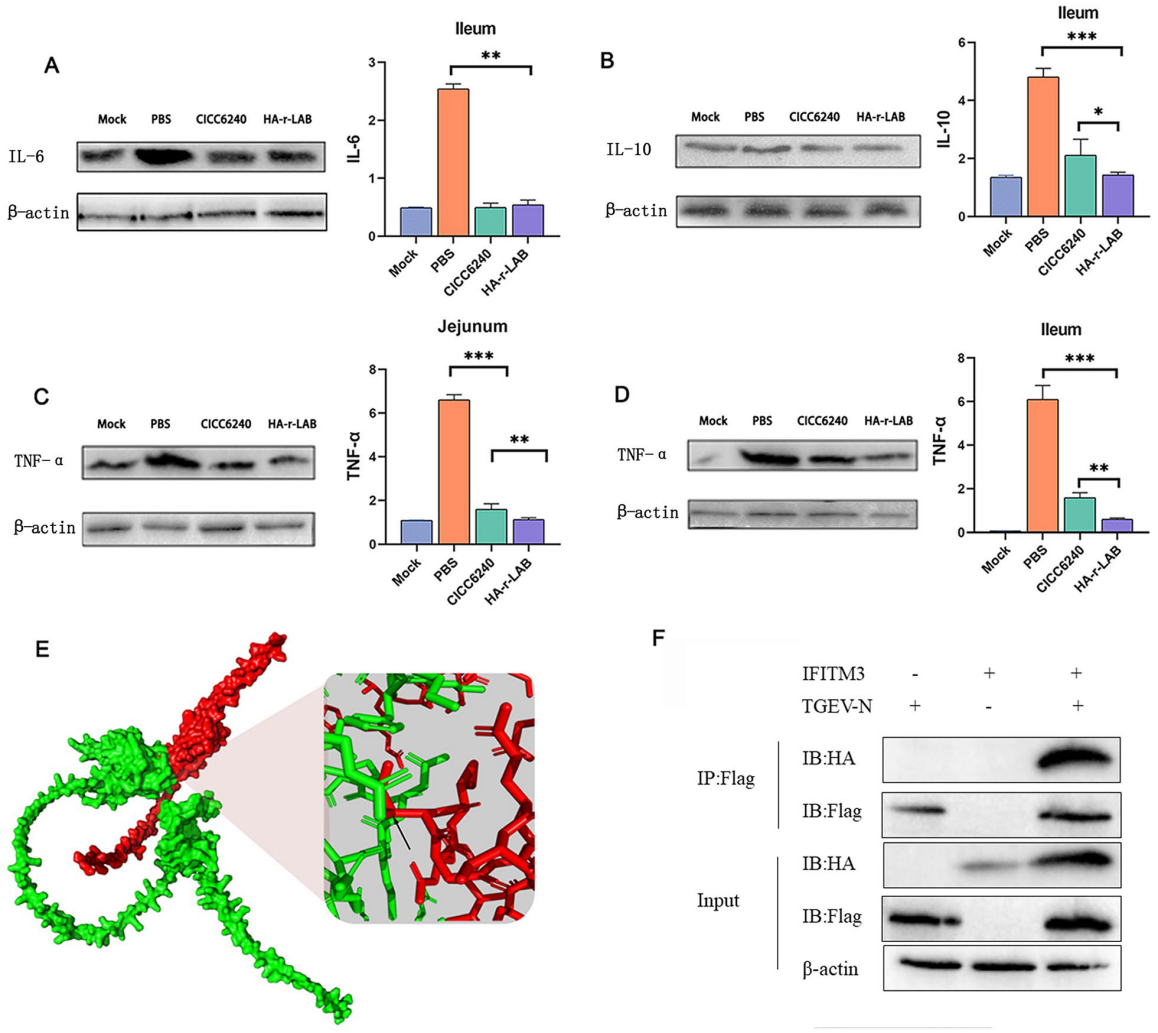
**Analysis of inflammatory cytokine expression and IFITM3-TGEV interaction in TGEV-infected piglets.** **A**–**D** Western blot analysis and corresponding bar graphs showing the protein expression levels of IL-6, IL-10, and TNF-α in the ileum and jejunum of piglets across different treatment groups: mock (uninfected control), PBS (phosphate-buffered saline-treated), CICC6240 (treated with the CICC6240 strain), and HA-r-LAB (treated with hyaluronic acid-encapsulated recombinant LAB). Red indicates the IFITM3 protein, whereas green indicates the TGEV-N protein. These results indicate that HA-r-LAB treatment significantly reduces the expression of these inflammatory cytokines compared with those in the PBS and CICC6240 groups. **E** 3D structural model of the TGEV-N protein, providing insights into its potential interactions with host proteins. **F** Immunoprecipitation (IP) assay demonstrating the interaction between IFITM3 and TGEV-N. The presence of both IFITM3 and TGEV-N in the IP:Flag and IP:HA lanes confirms their interaction. The input control shows the presence of both proteins in the samples. The binding site is marked with a black arrow. The data were repeated three or more times and are expressed as the means ± SDs. *p < 0.05; **p < 0.01; ***p < 0.001.

Protein expression analyses revealed that HA-r-LAB treatment significantly reduced IL-6 levels in the ileum compared with those in the PBS group (Figure 5A). Moreover, IL-10 protein expression was lower in the HA-r-LAB group than in both the PBS and CICC6240 groups (Figure 5B), and similar decreases were observed for TNF-α (Figures 5C–D). These results demonstrate that HA-r-LAB effectively suppresses the expression of both pro-inflammatory (IL-6, TNF-α) and anti-inflammatory (IL-10) cytokines, thereby mitigating the inflammatory response.

To further elucidate the underlying mechanisms, we utilized AlphaFold3 for protein interaction modelling, which predicted an interaction between the TGEV nucleocapsid (N) protein and IFITM3. This interaction was subsequently confirmed via immunoprecipitation, which demonstrated that IFITM3 binds to TGEV (Figure 5E). This novel finding suggests that IFITM3 may directly enhance host defense against TGEV by interfering with viral activity; thus, IFITM3 is a promising therapeutic target (Figure 5F).

### Transcriptome analysis of the TGEV-N protein

**Figure 6:**
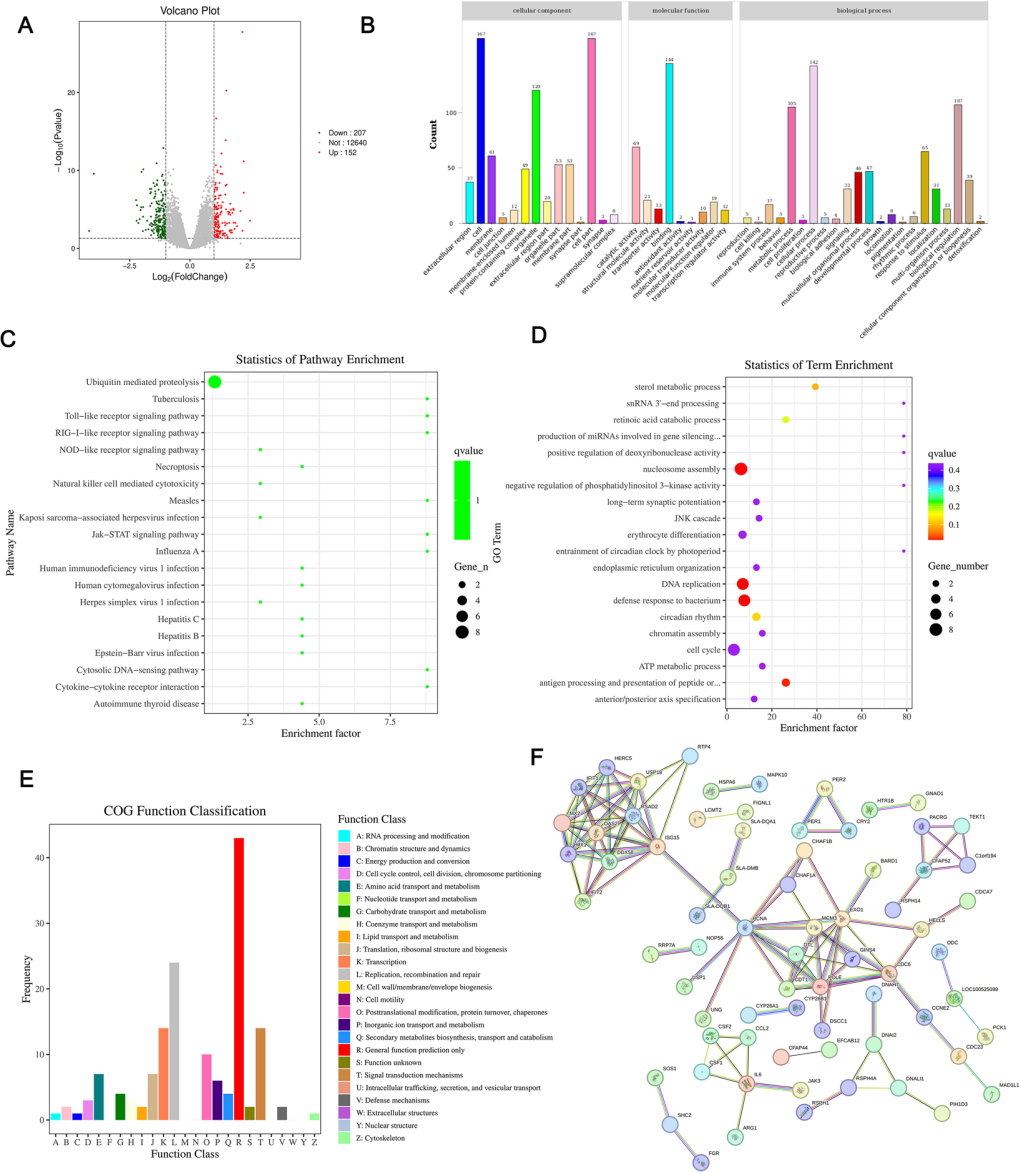
**Comprehensive transcriptome analysis of TGEV-N-infected cells.** **A** Volcano plot illustrating differential gene expression upon TGEV-N treatment, highlighting significantly upregulated (red dots) and downregulated (green dots) genes compared with the adjusted *p* value and fold change thresholds. **B** Bar charts depicting the number of DEGs across various molecular functions and biological processes categorized by gene ontology (GO), emphasizing the impact of TGEV-N on host cell functions. **C** Pathway enrichment analysis showing the enrichment factors of various pathways affected by TGEV-N, with a focus on those with significant enrichment scores, indicating potential disruption of these pathways. **D** Term enrichment statistics for differentially expressed genes, displaying the enrichment factor and corresponding gene count for each term, with color coding for the p value denoting significance. **E** COG (Clusters of Orthologous Groups) function classification bar chart, detailing the proportion of genes assigned to different functional categories, providing insights into the cellular processes affected by TGEV-N. **F** Gene coexpression network graph, where nodes represent genes and edges indicate significant coexpression relationships, highlighting the interconnectedness of genes within the network, which may play a role in TGEV-N-mediated responses. This figure collectively presents a multifaceted view of the molecular changes induced by TGEV-N in host cells, offering a foundation for understanding virus‒host interactions and potential therapeutic targets.

Comprehensive transcriptome sequencing was conducted to assess the impact of the TGEV-N protein on host gene expression. The analysis revealed that TGEV-N significantly altered the expression of 152 genes (upregulated) and 207 genes (downregulated) (Figure 6A), indicating extensive modulation of cellular processes.

Gene Ontology (GO) enrichment analysis indicated that genes involved in stimulus response (28%), metabolic regulation (22%), and transcriptional control (18%) were predominantly affected (Figure 6B). KEGG pathway analysis highlighted disruptions in immune-related pathways such as tuberculosis (*p* = 3.2 × 10^–5^) and Toll-like receptor signalling (*p* = 1.8 × 10^–4^) (Figure 6C). Additionally, term-specific enrichment identified the cell cycle (enrichment factor = 4.7) and DNA replication (enrichment factor = 3.9) as key processes (Figure 6D).

COG classification further revealed that energy metabolism (24%), amino acid transport (19%), and carbohydrate metabolism (15%) were major targets (Figure 6E), suggesting that TGEV-N induces metabolic reprogramming. Co-expression network analysis revealed tightly interconnected gene clusters (average clustering coefficient = 0.83), particularly within the immune regulation and apoptosis modules (Figure 6F).

These multiomics findings indicate that the TGEV-N protein hijacks the core cellular machinery, disrupting immune defenses, altering metabolic flux, and compromising genomic stability. These findings provide valuable mechanistic insights into TGEV pathogenesis and highlight potential antiviral targets.

### HA-r-LAB reduces TGEV viral load in piglet tissues

**Figure 7:**
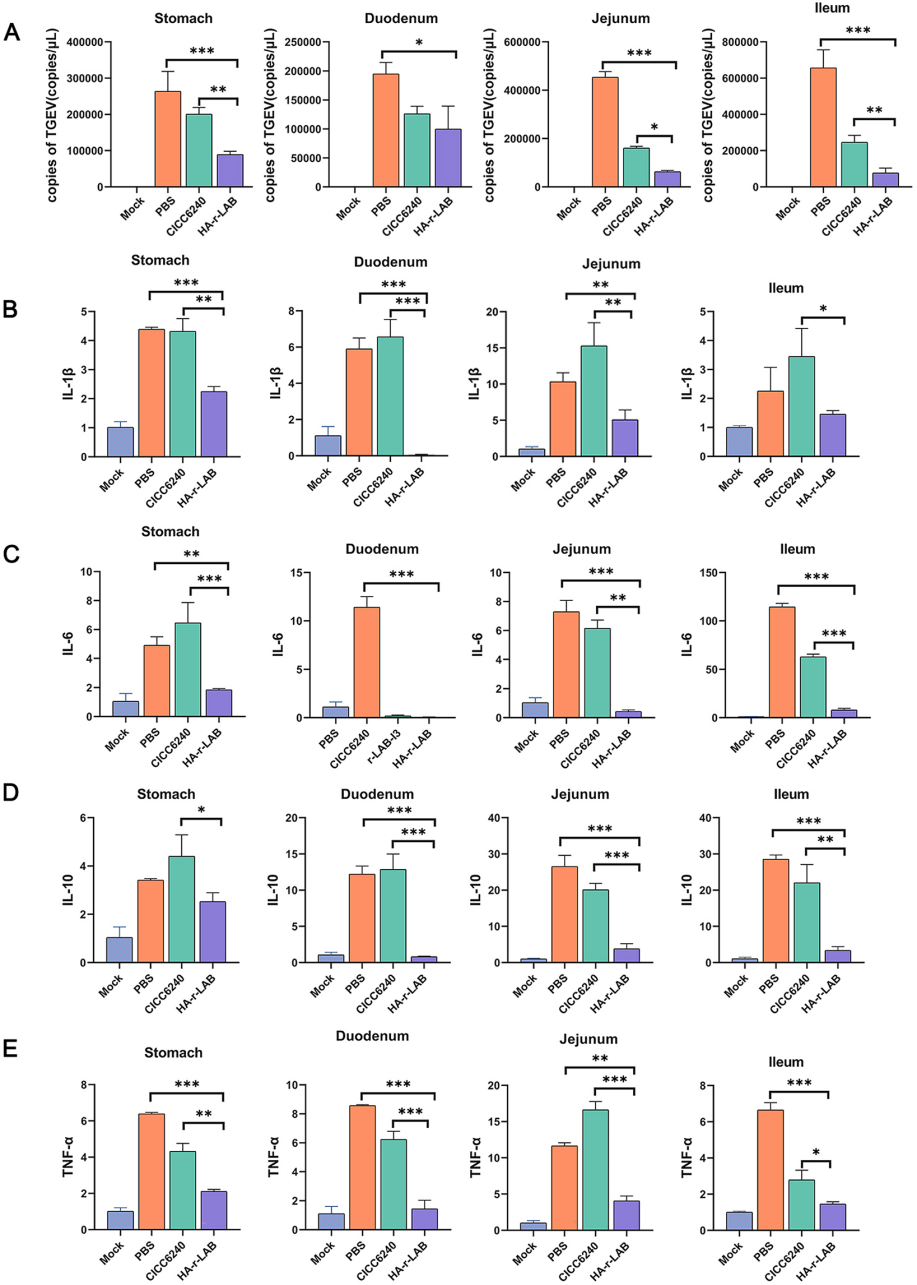
**Impact of treatment regimens on inflammatory cytokine levels and TGEV load in piglets.** **A** Bar graphs showing the number of TGEV per milliliter of tissue homogenate in the stomach, duodenum, jejunum, and ileum across different treatment groups: mock (uninfected control), PBS (phosphate-buffered saline-treated), CICC6240 (treated with the CICC6240 strain), and HA-r-LAB (treated with hyaluronic acid-encapsulated recombinant LAB). Significant reductions in TGEV load were observed in the HA-r-LAB group compared with the other groups. **B**–**E** Bar graphs illustrating the relative expression levels of the inflammatory cytokines IL-1β, IL-6, IL-10, and TNF-α in the stomach, duodenum, jejunum, and ileum. The expression levels were significantly lower in the HA-r-LAB group than in the PBS and CICC6240 groups, indicating a reduced inflammatory response. The data were repeated three or more times and are expressed as the means ± SDs. *p < 0.05; **p < 0.01; ***p < 0.001.

To further validate our western blot (WB) and transcriptomic results, we conducted quantitative polymerase chain reaction (qPCR) assays on piglet tissues. Using a TGEV standard plasmid assay (R^2^ = 0.996), viral loads were quantified in the stomach, duodenum, jejunum, and ileum. Compared with the PBS and CICC6240 groups, the HA-r-LAB group presented a significant reduction in TGEV load, with the mock group showing the lowest TGEV load (Figure 7A). Similar trends were observed in the duodenum and jejunum, where HA-r-LAB treatment resulted in the lowest viral loads among all the groups. In the ileum, the HA-r-LAB group again presented a significant decrease in the number of viral copies, whereas the number of viral copies in the PBS group approached 800 000.

### HA-r-LAB significantly reduces inflammatory cytokine transcription

The transcription levels of inflammatory cytokines were measured in the stomach, duodenum, jejunum, and ileum. The PBS and CICC6240 groups presented the highest IL-1β levels across all the tissues. Although the IL-1β level in the ileum of the HA-r-LAB group was not significantly different from that in the PBS group, the levels in the duodenum and stomach were lower (Figure 7B). Similarly, IL-6 transcription was significantly lower in the mock and HA-r-LAB groups than in the PBS and CICC6240 groups (Figure 7C). Although the level of IL-10 in the stomach was not significantly different, the HA-r-LAB group presented markedly lower levels in the duodenum, jejunum, and ileum (Figure 7D). The TNF-α levels in the stomach, duodenum, and ileum were also significantly reduced in the HA-r-LAB group. Overall, HA-r-LAB treatment significantly decreased the transcription of IL-1β, TNF-α, IL-6, and IL-10 in piglet tissues.

## Discussion

In this study, we successfully constructed a recombinant *L. plantarum* strain (r-LAB) expressing porcine IFITM3 and further encapsulated it with hyaluronic acid to increase its biological stability and delivery efficiency (HA-r-LAB). As illustrated in Figure 1, HA-r-LAB were designed to deliver IFITM3 orally to the intestinal mucosa, where the protein is absorbed by epithelial cells and interferes with TGEV replication via interaction with the TGEV-N protein. This delivery system effectively exploits the mucosal immune interface of the gastrointestinal tract, offering a promising vehicle for antiviral protein therapeutics.

Transcriptomic analyses revealed extensive host gene regulation in response to TGEV-N protein expression. Notably, interferon-stimulated genes (ISGs), such as ISG15 and MX1, were significantly upregulated, which is consistent with previous reports that TGEV infection initially induces ISG expression [30]. However, accumulating evidence indicates that TGEV suppresses ISG production at later stages of infection [31], thereby facilitating viral replication. This dynamic regulation aligns with findings that the induction of type I interferon and ISG expression in MARC-145 cells can effectively restrict porcine epidemic diarrhea virus (PEDV) replication [32], suggesting that evasion of this innate immune response may constitute a common immune escape strategy among swine enteric coronaviruses. Furthermore, the expression of proinflammatory cytokines, including IL-6, TNF-α, and IL-1β, was upregulated, which is in agreement with earlier findings that TGEV infection induces a cytokine-rich inflammatory milieu that contributes to mucosal injury [33]. These results reinforce the critical role of IFITM3, a well-characterized ISG with broad antiviral activity [21, 34, 35], in restoring interferon-mediated defense while simultaneously modulating virus-induced hyperinflammation.

SEM analysis provided clear morphological evidence that hyaluronic acid (HA) formed a stable and continuous coating around *L. plantarum*, confirming successful encapsulation. Importantly, this coating did not impair bacterial growth kinetics, suggesting that HA is biologically compatible with the probiotic host. These findings are consistent with an earlier report that HA-based encapsulation materials can protect probiotics without interfering with their intrinsic metabolic activity [36]. In addition to maintaining viability, HA encapsulation markedly enhanced bacterial resistance to simulated gastrointestinal fluids and promoted greater survival in different intestinal segments, which is in line with previous studies showing that HA or related polysaccharide coatings improve acid–bile tolerance and facilitate intestinal colonization [37, 38]. Collectively, these results highlight that HA encapsulation not only ensures the structural stability and physiological fitness of engineered bacteria but also provides a functional advantage by supporting their persistence and effective mucosal delivery in vivo.

In vivo experiments in TGEV-infected piglets demonstrated that HA-r-LAB markedly improved survival rates and alleviated intestinal pathology compared with those in the control groups. These results align with previous findings in which recombinant Lactobacillus strains expressing antiviral antigens conferred potent protection against TGEV infection. For example, oral administration of *L. plantarum* expressing the TGEV spike protein significantly enhances mucosal immunity against TGEV [39]. These parallels underscore the robust therapeutic potential of recombinant probiotic delivery systems in combating TGEV. The observed reductions in viral loads and inflammatory cytokine expression across intestinal segments further support the dual antiviral and anti-inflammatory activities of HA-r-LAB. Nevertheless, this study was limited to a single-pathogen infection model. Given that multifactorial enteric infections (e.g., TGEV/PEDV coinfection) are more common in pig production, future studies should evaluate HA-r-LAB under these complex conditions to better simulate field applications.

In conclusion, this investigation delineates critical accomplishments, including i. the successful development of a recombinant Lactobacillus strain encapsulated in hyaluronic acid (HA-r-LAB) engineered to deliver the porcine-derived IFITM3 protein to the intestinal mucosa; ii. The demonstration that HA-r-LAB significantly mitigate TGEV infection in vivo by integrating viral suppression, immune modulation, and tissue protection; iii. HA-r-LAB effectively reduced the transcription and expression of inflammatory cytokines in TGEV-infected piglets. Collectively, these findings underscore HA-r-LAB as a promising mucosal delivery platform for antiviral proteins and lay the foundation for the development of novel immunotherapeutic approaches in veterinary medicine.

## Data Availability

The datasets generated and/or analyzed during the current study are available from the corresponding author upon reasonable request.
